# Public attitudes and knowledge about self‐injury: A cross‐sectional web‐based survey of Japanese adults

**DOI:** 10.1002/pcn5.70033

**Published:** 2024-11-05

**Authors:** Masaru Takahashi, Kasumi Imahara, Yukiko Miyamoto, Kayoko Myojo, Michiko Yasuda, Izumi Kadomodo

**Affiliations:** ^1^ Faculty of Core Research, Ochanomizu University Tokyo Japan; ^2^ Osaka Juvenile Classification Home, Ministry of Justice Osaka Japan; ^3^ Nagoya Juvenile Classification Home, Ministry of Justice Nagoya Japan; ^4^ Kyoto Juvenile Classification Home, Ministry of Justice Kyoto Japan; ^5^ Osaka Prison, Ministry of Justice Osaka Japan; ^6^ Department of Clinical Psychology Taisho University Tokyo Japan

**Keywords:** Japan, myth, nonsuicidal self‐injury, public attitude, suicide prevention

## Abstract

**Aim:**

Nonsuicidal self‐injury is common among adolescents. Dispelling related public misconceptions is essential for creating effective prevention and intervention strategies, as these myths contribute to stigmatization. This study examined the prevalence of self‐injury myths among the Japanese public and explored how demographics and personal experiences influence these beliefs.

**Methods:**

A nationwide web‐based self‐report survey of 2000 Japanese adults (mean age = 44.6 ± 14.3 years) examined their agreement with 14 scientifically unsupported self‐injury beliefs. Endorsement rates for each myth were calculated, and the associations between myth beliefs and other variables were explored.

**Results:**

Fourteen myths were analyzed, with endorsement rates of 21.0%–68.7%. Logistic regression analysis indicated that men were more likely to endorse the myth that self‐injury is uncommon (adjusted odds ratio [AOR] = 1.45, 95% confidence interval [CI] = 1.17–1.79), whereas women were more prone to myths about the rarity of self‐injury, using a single method, stimulation as the exclusive purpose, wrist‐cutting prevalence, and average onset age. Younger participants were more likely to believe that self‐injury is solely for stimulation than other age groups. Those with human‐service professional experience were more likely to view self‐injury as attention‐seeking compared to those without such experience (AOR = 1.62, 95% CI = 1.07–2.46). With some exceptions, individuals who expressed confidence in their ability to respond to self‐injurers were more likely to endorse the myths.

**Conclusion:**

These findings highlight the need for targeted educational interventions to dispel misconceptions about self‐injury and improve public understanding of this complex behavior.

## INTRODUCTION

Nonsuicidal self‐injury (NSSI) is a common behavior, particularly among adolescents, and constitutes a key public health concern. A recent meta‐analysis including 439,818 participants reported a 16% NSSI prevalence rate among adolescents between 2015 and 2020, indicating that one in six adolescents had a history of self‐injury.^1^ Another review found a high prevalence of NSSI in nonclinical samples of adolescents, indicating an aggregate lifetime prevalence of 22% in children and adolescents.^2^ Indeed, prevalence estimates may vary depending on population characteristics, assessment methodologies, and operational definitions employed; however, it is evident that NSSI is a widespread phenomenon among youth. Gender discrepancies have been observed in the prevalence of NSSI, with women typically exhibiting higher rates than men.^1^ However, gender differences in NSSI are not static, with the gap widening during mid‐adolescence and disappearing by early adulthood.^3^ Moreover, the gender disparity is more pronounced in clinical populations than in college or community samples.^4^

An accurate understanding of the prevalence, correlates, and functions of NSSI is crucial for developing effective suicide prevention and intervention strategies as individuals with a history of self‐injury are at a higher risk of attempting suicidal behaviors later in life.^5^, ^6^, ^7^, ^8^, ^9^, ^10^ However, there is a widespread presence of inaccurate beliefs about suicide and self‐injury.^11^ For instance, common myths about suicide include the following: people who talk about suicide do not really want to die; people who talk about suicide just seek attention; suicides always occur without warning; talking about suicide increases the chance that a person will act on it; teenagers present the highest risk of suicide; and, if a person intends to attempt suicide, there is no way to stop it.^12^

Demographic factors and personal experience may influence these myths. A study conducted on a sample of Australian adults found that being male, speaking a language other than English at home, and being older than 60 years of age were significantly associated with the strongest beliefs in suicide myths.^13^ Lower exposure to suicide, higher age, being male, lower education, and culturally diverse backgrounds were also linked to lower suicide literacy.^14^ A population survey from Germany revealed that suicide literacy was negatively associated with age and positively associated with education, affliction, and personal contact.^15^

As with suicide, the public holds a variety of misconceptions about self‐injury. Despite the widespread misconception that self‐injury predominantly affects women, empirical evidence suggests that the lifetime prevalence of self‐injury in men is higher than assumed, although not as high as in women, and the gender gap is narrowing, especially in nonclinical populations.^4^ Adherence to this false belief may lead to a lack of adequate understanding and support for men who self‐injure. Another prevalent myth is that self‐injury is employed solely to seek attention. Contrary to the assertion that self‐injury is primarily aimed at influencing others, research consistently demonstrates that NSSI is initially conducted in isolation, not in public, and that NSSI serves multiple functions, with emotion regulation being the most prominent.^16^, ^17^, ^18^ Adherence to this erroneous belief can lead to adverse consequences, such as stigmatization and a lack of support, for those who engage in it.^19^ There is also a widespread misconception that self‐injury is a form of suicide attempt. However, self‐injury can manifest independently of suicidal ideation, and the two phenomena are fundamentally distinct in their intent and purpose.^14^ Self‐injury involves the deliberate infliction of harm on oneself as a means of managing emotional distress. The primary objective is not to terminate one's life but rather to alleviate unbearable pain and emotional numbing, with common methods including cutting, severe scratching, burning, banging, and hitting.^20^ Conversely, suicide attempts are actions undertaken with the explicit intent to end one's life, employing lethal methods. Individuals who attempt suicide often experience profound hopelessness and a sense of entrapment, perceiving no alternative means to alleviate their suffering. While self‐injury should not be considered inconsequential, as it elevates the risk of future suicide in the long term,^21^ equating both acts as having precisely the same purpose and intent may result in an inappropriate response.

Dispelling myths about self‐injury is crucial, as such misconceptions can be conveyed to those who self‐injure, contributing to public stigma that may deter them from seeking support. Despite extensive research on suicide myths, there is limited research on self‐injury myths in the general population rather than professionals. Moreover, even fewer studies have comprehensively examined individuals' perceptions of a broad range of myths as opposed to addressing them individually. Therefore, evidence and data are lacking on the prevalence of myths and misconceptions about self‐injury and their relationship with demographic factors, personal experiences, and beliefs. Moreover, the active participation of the public is essential for implementing effective suicide prevention policies. Additionally, while attitudes and knowledge of self‐injury have been examined with different practitioners in various settings—including school teachers,^22^ prison officers,^23^ emergency and mental health nurses,^24^ and certified mental health professionals^25^—the public's perspectives have received less attention. Promoting suicide prevention measures requires the support and cooperation of a diverse range of individuals. Determining the extent of the public's misconceptions about self‐injury and identifying the specific personal experiences and sociodemographic characteristics associated with these myths may aid in targeting suicide prevention education efforts. Additionally, previous research on suicide myths has primarily been conducted in Western countries, indicating the scarcity of studies in non‐Western societies. Given the role of culture in the emergence and stigmatization of self‐injury, it is essential to investigate public beliefs about self‐injury in non‐Western countries to gain new insights and enhance the current knowledge in this field.

This study addresses these knowledge gaps by investigating the prevalence of myths and misconceptions about self‐injury among the Japanese public, and by examining the relationship between demographic factors, personal experiences, and beliefs in myths.

## METHODS

### Participants

Participants were 2000 Japanese adults, including 1000 men and 1000 women, recruited through the online research company Cross Marketing, Inc. The company's database comprises more than 10 million individuals, constituting a considerable and varied sample of Japan's demographics encompassing a range of ages and backgrounds. After an initial screening to eliminate respondents who were not sufficiently careful in their responses, as described below, recruitment continued until the target number of 200 participants per age group (20, 30, 40, 50, and 60 s) and gender was reached. Sample size calculations based on power and effect size were not performed. Participants' mean age was 44.6 ± 14.3 years (range 20–69 years).

### Procedure

Data were collected between December 15 and 17, 2023. The web‐based survey began with a clear statement that participation was voluntary and that individuals could withdraw at any point. Furthermore, participant anonymity was guaranteed; the submission of their responses was considered as giving consent to participate. During the recruitment process, three control questions were randomly presented throughout the survey, instructing participants to select a specific response option, such as “This is a control question. Select ‘somewhat not applicable’ for this question.” These questions were designed to identify participants who may have been inattentive or failed to allocate sufficient cognitive resources during the response process.^23^ Respondents who provided incorrect answers to any of those questions were excluded from the survey. The total number of individuals excluded from this study was 276, 225, 226, 197, and 270 in their 20s, 30s, 40s, 50s, and 60s, respectively.

### Measures

#### Self‐injury myths

A literature review was conducted to identify existing measures of attitudes toward self‐injury and articles pertaining to self‐injury myths. No suitable measure aligning with the study objectives was found. Consequently, we identified several commonly believed but scientifically unsubstantiated discourses utilizing previous research, websites of academic societies about self‐injury, and the authors' clinical experiences and designated them as myths. Following the administration of the questionnaire, the items that lacked sufficient scientific basis to be categorized as myths or those that may be misconstrued owing to inaccurate phrasing were eliminated from the analysis. The remaining 14 items were selected for further analysis: (1) “self‐injury is an uncommon phenomenon”; (2) “most students who self‐injure are girls”; (3) “self‐injury, including wrist‐cutting, is a form of attempted suicide”; (4) “self‐injury is exclusively undertaken for stimulation”; (5) “people who self‐injure have a mental disorder”; (6) “people who self‐injure are attention‐seekers”; (7) “self‐injury is specific to those who are withdrawn and depressed”; (8) “habitual self‐injurers usually use only one method of self‐injury”; (9) “self‐injurious behavior consists mainly of wrist‐cutting”; (10) “people who self‐injure do it more to influence others than to change something in themselves”; (11) “the average onset age of self‐injury is approximately 16 years”; (12) “very few minors disclose their experiences of self‐injury to friends and acquaintances”; (13) “self‐injury is driven by the desire to be cared about; therefore, it will decrease if ignored”; and (14) “talking about self‐injury encourages it.”

For each myth, we provide empirical evidence to refute this. For instance, concerning the first myth in the above list, community prevalence studies indicate that self‐injury is a widespread phenomenon, particularly among adolescents and young adults,^1^, ^2^ contrary to the notion that self‐injury is uncommon. Similarly, for other myths, those with empirical research findings that refute them were selected. Participants indicated their agreement with the myths using a six‐point Likert scale ranging from 1 (strongly disagree) to 6 (strongly agree).

#### Experience related to self‐injury

Respondents answered the following questions regarding suicide and self‐injury with either “yes” or “no”: having (or had) family members, friends, or acquaintances who repeatedly self‐injure(d) and having worked as a human‐service professional (e.g., doctor, nurse, teacher, psychologist, counselor). Furthermore, participants were asked whether they were confident in their ability to respond appropriately if someone around them engaged in self‐injury.

#### Demographic variables

The survey included inquiries about respondents' gender, age, and marital and parental status.

### Statistical analysis

To gauge the degree of endorsement of self‐injury myths, we reclassified the responses from the six‐point Likert scale into binary categories of agreement or disagreement. This allowed us to gain a more comprehensive understanding of the sample. Subsequently, we calculated the percentage of endorsements for each myth by gender. Chi‐squared tests were employed to analyze gender differences in support of each myth, with P‐values adjusted using the Bonferroni correction for multiple comparisons. The adjusted P‐value was set at 0.0036 (= 0.05/14) as 14 comparisons were conducted. Following this, logistic regression analyses were conducted to investigate the association between sociodemographic and related experience variables, and beliefs in these myths. All statistical analyses were performed using SPSS version 29.

## RESULTS

### Descriptive statistics of participants and their endorsement of each myth by gender

Table 1 presents participants’ descriptive statistics. As depicted in Table 1, 13.5% of all participants indicated that they had, or had had, a family member, friend, or acquaintance who repeatedly engaged in self‐injury. Of the total respondents, 9.1% expressed confidence in their ability to cope with self‐injury effectively. Moreover, approximately 5% of all respondents reported that they had been or were currently professionally involved in human services‐related fields.

**Table 1:** Participant sociodemographic characteristics.

	Men	Women	Total
	(*n* = 1000)	(*n* = 1000)	(*N* = 2000)
Married (including separated or bereaved)	42.9	61.0	52.0
Having children	33.0	44.4	38.7
Experience of being a human‐service professional	4.2	6.3	5.3
Having family or friends who repeatedly engaged in self‐injury	10.7	16.2	13.5
Confidence to respond appropriately to people who self‐injure	10.4	7.8	9.1
Feelings of unforgiveness toward self‐injurers	27.3	21.0	24.2

Table 2 shows the endorsement rates for each myth by gender. The most endorsed myth in this study was “very few minors disclose their experiences of self‐injury to friends and acquaintances,” with 68.7% of the participants supporting this statement. Following closely behind, 68.3% of the participants agreed with the statement “self‐injury, including wrist‐cutting, is a form of attempted suicide.” Additionally, nearly half the respondents believed that “self‐injurious behavior consists mainly of wrist‐cutting,” and “people who self‐injure have a mental disorder.” The least supported myth was “self‐injury is driven by the desire to be cared about; therefore, it will decrease if ignored,” with only 21.0% of the participants endorsing it. Regarding gender, only the myth that “self‐injury is an uncommon phenomenon” demonstrated a significant difference in the proportion of men compared to women who endorsed it, following the adjustment of P‐values using Bonferroni correction.

**Table 2:** Percentage of self‐injury myth endorsers by gender.

	Men	Women	Total	P
	(*n* = 1000)	(*n* = 1000)	(*N* = 2000)	
Very few minors disclose their experiences of self‐injury to friends and acquaintances	66.6	70.8	68.7	0.043
Self‐injury, including wrist‐cutting, is a form of attempted suicide	67.5	69.1	68.3	0.442
Self‐injurious behavior consists mainly of wrist‐cutting	48.3	54.4	51.4	0.006
People who self‐injure have a mental disorder	48.6	49.1	48.9	0.823
People who self‐injure are attention‐seekers	39.5	42.1	40.8	0.237
Habitual self‐injurers usually use one method of self‐injury	35.2	40.3	37.8	0.019
The average onset age of self‐injury is approximately 16 years	34.6	40.9	37.8	0.004
Most students who self‐injure are girls	33.2	35.3	34.3	0.322
People who self‐injure do it more to influence others than to change something in themselves	32.8	33.2	33.0	0.849
Self‐injury is specific to those who are withdrawn and depressed	29.3	30.6	30.0	0.526
Talking about self‐injury encourages it	29.2	25.8	27.5	0.089
Self‐injury is an uncommon phenomenon	28.4	20.2	24.3	<0.001
Self‐injury is exclusively undertaken for stimulation	23.1	25.6	24.3	0.193
Self‐injury is driven by the desire to be cared about; therefore, it will decrease if ignored	20.9	21.1	21.0	0.913

*Note*: The P‐values presented in this table represent the values before adjustment using the Bonferroni correction. Because a 14‐pair comparison was being conducted, establishing the P‐value threshold for significance at 0.05 results in a corrected significance level of 0.0036.

### Logistic regression analyses targeting endorsement of each myth

Table 3 presents the results of the logistic regression analysis, with belief in each myth serving as the dependent variable, while the independent variables were gender, age group, experience of having family members, friends, or acquaintances who repeatedly engaged in self‐injury, professional experience in human‐service, confidence in appropriately responding to self‐injurers, and believing that self‐injury is unacceptable. The adjusted odds ratio (AOR) for gender was significant and >1 for the statement that “self‐injury is an uncommon phenomenon” (AOR = 1.45, 95% confidence interval [CI] = 1.17–1.79), indicating that men were more likely to believe this myth than women after controlling for other confounding variables. In contrast, the AORs for sex were significant and <1 for the following five statements indicating that more women than men believed these myths: “habitual self‐injurers usually use one method of self‐injury” (AOR = 0.77, 95% CI = 0.64–0.92), “self‐injury is exclusively undertaken for stimulation” (AOR = 0.77, 95% CI = 0.62–0.96), “self‐injurious behavior consists mainly of wrist‐cutting” (AOR = 0.75, 95% CI = 0.63–0.90), “the average onset age of self‐injury is approximately 16 years” (AOR = 0.74, 95% CI = 0.61–0.88), and “very few minors disclose their experiences of self‐injury to friends and acquaintances” (AOR = 0.80, 95% CI = 0.66–0.97).

**Table 3:** Multiple logistic regression analysis using demographic factors to predict myth endorsement.

	Self‐injury, including wrist‐cutting, is a form of attempted suicide				Self‐injury is an uncommon phenomenon			
	OR	95% CI	AOR	95% CI	OR	95% CI	AOR	95% CI
Gender (male)	0.93	0.77–1.12	0.90	0.74–1.09	1.57***	1.27–1.93	1.45***	1.17–1.79
Age, years								
20–29 (ref.)	1.00	–	1.00	–	1.00	–	1.00	–
30–39	0.76	0.56–1.02	0.76	0.56–1.02	1.00	0.72–1.39	1.04	0.74–1.46
40–49	0.77	0.57–1.03	0.77	0.57–1.04	1.07	0.77–1.49	1.11	0.79–1.56
50–59	0.90	0.66–1.22	0.88	0.65–1.20	1.16	0.84–1.61	1.16	0.83–1.62
60–69	0.91	0.67–1.23	0.87	0.64–1.18	1.31	0.95–1.80	1.24	0.89–1.74
Family or friends who repeatedly engaged in self‐injury	1.03	0.78–1.35	1.02	0.76–1.36	0.61**	0.43–0.85	0.63**	0.44–0.89
Human‐service professional	0.97	0.64–1.47	0.95	0.62–1.47	0.77	0.47–1.25	0.78	0.46–1.31
Confidence to respond appropriately	1.08	0.78–1.50	0.93	0.66–1.31	2.11***	1.53–2.90	1.88***	1.34–2.64
Feelings of unforgiveness	1.79***	1.41–2.27	1.81***	1.42–2.31	2.29***	1.83–2.86	2.07***	1.64–2.60

	Most students who self‐injure are girls				People who self‐injure have a mental disorder			
	OR	95% CI	AOR	95% CI	OR	95% CI	AOR	95% CI
Gender (male)	0.91	0.76–1.10	0.89	0.74–1.07	0.98	0.82–1.17	0.92	0.77–1.10
Age, years								
20–29 (ref.)	1.00	–	1.00	–	1.00	–	1.00	–
30–39	0.97	0.73–1.29	1.00	0.75–1.34	0.99	0.75–1.31	1.03	0.78–1.37
40–49	0.83	0.62–1.11	0.86	0.64–1.16	0.91	0.69–1.21	0.96	0.72–1.27
50–59	0.83	0.62–1.11	0.86	0.64–1.16	0.90	0.68–1.18	0.91	0.69–1.21
60–69	0.87	0.65–1.16	0.87	0.65–1.17	0.82	0.61–1.06	0.78	0.59–1.04
Family or friends who repeatedly engaged in self‐injury	1.34*	1.03–1.75	1.23	0.94–1.62	1.08	0.84–1.40	1.07	0.82–1.41
Human‐service professional	1.41	0.95–2.10	1.21	0.80–1.83	0.74	0.50–1.11	0.65*	0.42–0.98
Confidence to respond appropriately	1.78***	1.31–2.42	1.55**	1.13–2.13	1.90***	1.39–2.61	1.69**	1.22–2.34
Feelings of unforgiveness	1.56***	1.26–1.92	1.51***	1.22–1.87	2.03***	1.65–2.51	2.00***	1.61–2.47

	People who self‐injure are attention‐seekers				Self‐injury is specific to those who are withdrawn and depressed			
	OR	95% CI	AOR	95% CI	OR	95% CI	AOR	95% CI
Gender (male)	0.90	0.75–1.07	0.85	0.70–1.02	0.94	0.78–1.14	0.88	0.72–1.07
Age, years								
20–29 (ref.)	1.00	–	1.00	–	1.00	–	1.00	–
30–39	1.06	0.80–1.41	1.07	0.80–1.42	0.87	0.65–1.16	0.91	0.68–1.23
40–49	0.94	0.71–1.25	0.96	0.72–1.28	0.71*	0.53–0.96	0.75	0.55–1.01
50–59	0.69*	0.52–0.92	0.67**	0.50–0.90	0.61**	0.45–0.82	0.63**	0.46–0.86
60–69	0.87	0.65–1.15	0.79	0.59–1.05	0.70*	0.52–0.94	0.70*	0.51–0.95
Family or friends who repeatedly engaged in self‐injury	1.16	0.89–1.50	1.02	0.77–1.34	1.18	0.90–1.56	1.07	0.80–1.43
Human‐service professional	1.71**	1.15–2.53	1.62*	1.07–2.46	1.03	0.67–1.57	0.84	0.53–1.32
Confidence to respond appropriately	1.47*	1.08–2.00	1.12	0.81–1.55	2.71***	1.99–3.69	2.37***	1.72–3.26
Feelings of unforgiveness	2.47***	2.00–3.04	2.55***	2.05–3.16	1.87***	1.51–2.31	1.76***	1.41–2.20

	Habitual self‐injurers usually use one method of self‐injury				Self‐injury is exclusively undertaken for stimulation			
	OR	95% CI	AOR	95% CI	OR	95% CI	AOR	95% CI
Gender (male)	0.81**	0.67–0.96	0.77**	0.64–0.92	0.87	0.71–1.07	0.77*	0.62–0.96
Age, years								
20–29 (ref.)	1.00	–	1.00	–	1.00	–	1.00	–
30–39	1.11	0.84–1.48	1.17	0.87–1.57	0.58***	0.44–0.79	0.59***	0.44–0.81
40–49	0.94	0.70–1.25	0.99	0.73–1.33	0.38***	0.28–0.52	0.38***	0.27–0.53
50–59	1.22	0.92–1.63	1.28	0.96–1.72	0.30***	0.22–0.42	0.29***	0.21–0.41
60–69	1.20	0.90–1.60	1.22	0.91–1.63	0.28***	0.20–0.40	0.25***	0.17–0.35
Family or friends who repeatedly engaged in self‐injury	1.25	0.96–1.62	1.29	0.98–1.69	1.32	0.99–1.76	1.01	0.74–1.39
Human‐service professional	0.82	0.54–1.24	0.66	0.42–1.02	1.39	0.90–2.13	1.18	0.73–1.91
Confidence to respond appropriately	1.70***	1.25–2.30	1.58**	1.15–2.17	3.33***	2.44–4.55	2.60***	1.85–3.64
Feelings of unforgiveness	1.77***	1.44–2.19	1.73***	1.39–2.14	2.56***	2.05–3.20	2.67***	2.10–3.39

	Self‐injurious behavior consists mainly of wrist‐cutting				People who self‐injure do it more to influence others than to change something in themselves			
	OR	95% CI	AOR	95% CI	OR	95% CI	AOR	95% CI
Gender (male)	0.78**	0.66–0.93	0.75**	0.63–0.90	0.98	0.82–1.18	0.92	0.76–1.12
Age, years								
20–29 (ref.)	1.00	–	1.00	–	1.00	–	1.00	–
30–39	0.77	0.58–1.02	0.78	0.59–1.03	1.14	0.85–1.52	1.19	0.89–1.61
40–49	0.77	0.58–1.02	0.78	0.59–1.04	0.82	0.61–1.11	0.86	0.64–1.17
50–59	0.76	0.57–1.00	0.75	0.57–1.00	0.88	0.66–1.19	0.91	0.67–1.23
60–69	0.88	0.66–1.16	0.85	0.64–1.13	0.90	0.67–1.21	0.87	0.64–1.18
Family or friends who repeatedly engaged in self‐injury	1.15	0.89–1.48	1.14	0.87–1.49	1.26	0.97–1.65	1.17	0.89–1.56
Human‐service professional	0.79	0.53–1.17	0.72	0.48–1.09	1.32	0.88–1.98	1.11	0.72–1.71
Confidence to respond appropriately	1.12	0.82–1.52	0.98	0.71–1.34	2.14***	1.58–2.91	1.73***	1.25–2.39
Feelings of unforgiveness	1.76***	1.43–2.17	1.82***	1.47–2.26	2.44***	1.97–3.01	2.35***	1.89–2.92

	The average onset of self‐injury is approximately 16 years				Very few minors disclose their experiences of self‐injury to friends and acquaintances			
	OR	95% CI	AOR	95% CI	OR	95% CI	AOR	95% CI
Gender (male)	0.76**	0.64–0.92	0.74**	0.61–0.88	0.82*	0.68–0.99	0.80*	0.66–0.97
Age, years								
20–29 (ref.)	1.00	–	1.00	–	1.00	–	1.00	–
30–39	0.79	0.60–1.06	0.81	0.61–1.08	0.73*	0.54–0.99	0.73*	0.54–0.98
40–49	0.81	0.61–1.08	0.84	0.63–1.12	0.92	0.68–1.25	0.92	0.68–1.25
50–59	0.73**	0.55–0.97	0.74*	0.55–0.99	0.90	0.66–1.22	0.88	0.65–1.20
60–69	0.87	0.66–1.16	0.87	0.65–1.16	0.91	0.67–1.23	0.86	0.64–1.18
Family or friends who repeatedly engaged in self‐injury	1.14	0.88–1.49	1.03	0.78–1.36	1.07	0.81–1.41	1.05	0.78–1.40
Human‐service professional	1.25	0.84–1.86	1.10	0.73–1.67	1.04	0.68–1.60	1.03	0.66–1.61
Confidence to respond appropriately	1.83***	1.34–2.48	1.66**	1.21–2.28	0.95	0.68–1.31	0.82	0.58–1.15
Feelings of unforgiveness	1.48***	1.21–1.83	1.45***	1.17–1.79	1.67***	1.32–2.12	1.75***	1.38–2.23

	Self‐injury is driven by the desire to be cared about; therefore, it will decrease if ignored				Talking about self‐injury encourages it			
	OR	95% CI	AOR	95% CI	OR	95% CI	AOR	95% CI
Gender (male)	0.99	0.80–1.23	0.86	0.69–1.08	1.19	0.97–1.44	1.12	0.91–1.37
Age, years								
20–29 (ref.)	1.00	–	1.00	–	1.00	–	1.00	–
30–39	0.90	0.65–1.24	0.94	0.67–1.33	0.84	0.62–1.13	0.88	0.65–1.20
40–49	0.86	0.62–1.19	0.91	0.65–1.29	0.69*	0.51–0.93	0.73*	0.53–0.99
50–59	0.67*	0.47–0.94	0.66*	0.46–0.95	0.73*	0.54–0.98	0.75	0.55–1.03
60–69	0.70*	0.50–0.98	0.64*	0.45–0.91	0.55***	0.40–0.76	0.54***	0.39–0.74
Family or friends who repeatedly engaged in self‐injury	0.91	0.66–1.26	0.77	0.54–1.09	1.26	0.96–1.66	1.15	0.86–1.55
Human‐service professional	1.25	0.79–1.98	1.08	0.65–1.78	1.17	0.76–1.79	0.99	0.62–1.56
Confidence to respond appropriately	3.50***	2.55–4.80	2.82***	2.02–3.95	2.61***	1.92–3.56	2.12***	1.54–2.94
Feelings of unforgiveness	3.12***	2.48–3.93	2.95***	2.32–3.74	2.16***	1.74–2.69	2.06***	1.64–2.58

Abbreviations: AOR, adjusted odds ratio; CI, confidence interval; OR, crude odds ratio; ref. reference.

* P < 0.05

** P < 0.01

*** P < 0.001.

By age group, the myth that self‐injury is exclusively undertaken for stimulation was significantly less prevalent across all age groups, with a crude odds ratio < 1, compared to those in their 20s who served as the reference category. This finding suggests that this myth is more prevalent among individuals in their 20s than other among age groups. Similarly, the myth “self‐injury is driven by the desire to be cared about; therefore, it will decrease if ignored” was significantly less supported by those in their 50s and 60s than those in their 20s. Further comparative analyses utilizing individuals in their 20s as the reference group revealed significantly lower percentages of support for the myth “very few minors disclose their experiences of self‐injury to friends and acquaintances” among participants in their 30s, for the myths “the average onset age of self‐injury is approximately 16 years” and “people who self‐injure are attention‐seekers” among those in their 50s, and for the myth “talking about self‐injury encourages it” among individuals in their 40s and 60s. Regarding the other myths, no significant differences in endorsements by age group were found. Consequently, compared to other older age groups, several myths received greater support from individuals in their 20s, but not vice versa.

The presence of a family member or acquaintance who repeatedly engages in self‐injury was significantly associated only with the myth that self‐injury is an uncommon phenomenon, indicating that the greater the number of such family members, friends, and acquaintances present, the less likely individuals were to endorse this statement (AOR = 0.63, 95% CI = 0.44–0.89). Our findings indicate that individuals with experiences as human‐service professionals exhibit a significantly lower propensity to perceive self‐injury solely as a symptom of psychiatric disorder, compared to those without such experience (AOR = 0.65, 95% CI = 0.42–0.98). Conversely, a higher proportion of individuals with experience in human‐service professions endorsed the myth that people who self‐injure are attention‐seekers compared to those without such experience (AOR = 1.62, 95% CI = 1.07–2.46). Paradoxically, individuals who expressed confidence in their ability to respond adequately to individuals who engaged in self‐injury demonstrated significantly higher AOR of endorsing belief in 10 out of 14 myths presented in this study compared to those who did not. Furthermore, the results indicated that individuals who exhibited a lack of forgiveness toward self‐injurers demonstrated a higher propensity to endorse affirmative beliefs regarding all myths and held stronger convictions compared to those who did not report such attitudes.

## DISCUSSION

This study examined public attitudes and knowledge about self‐injury, exploring their relationship to gender, age, and prior experience. While belief in each myth varied, approximately 50%–70% of respondents supported four out of the 14 myths presented. Even the least supported myth, “self‐injury is driven by the desire to be cared about; therefore, it will decrease if ignored,” received agreement from more than 20% of participants. Additionally, certain myths showed significant associations with gender, age group, and individual experience.

Research shows that certain demographic groups, including males, older adults, and non‐English speakers, are more likely to endorse suicide myths.^13^ A significantly greater proportion of male participants believed that self‐injury is a rare phenomenon, despite empirical studies demonstrating that approximately 16%–22% of youth reported lifetime experiences.^1^, ^2^ Several potential factors may contribute to the higher proportion of men than women, perceiving self‐injury as an infrequent occurrence. First, the societal stigma surrounding masculinity and emotional expression may inhibit men from discussing or disclosing self‐injury, potentially leading to an underestimation of its prevalence. Cultural norms and gender stereotypes may also influence perceptions of mental health, associating self‐injury primarily with women and causing men to underestimate their occurrence in their gender. Additionally, media representations often focus on women's experiences of self‐injury, reinforcing the belief that it is less common among men.

The logistic regression analyses revealed that women were more likely than men to endorse several myths, even after controlling for potential confounders. For instance, women were more inclined to believe that habitual self‐injurers use a single method, despite evidence showing they often use various methods.^26^ Similarly, the myth that self‐injurious behavior consists mainly of wrist‐cutting was also more prevalent among women than men, aligning with research suggesting that women more frequently use cutting, whereas the prevalence of other methods, like punching, does not significantly differ between genders.^4^ Additionally, women were more likely to endorse the myth that most self‐injuring students were girls, contradicting research showing a substantial number of male students also engage in self‐injury. A meta‐analysis found women more likely to engage in NSSI, but the effect size was small, especially in community samples.^4^ Other research highlights significant self‐injury rates among male students, challenging the notion that it is primarily a “feminine” behavior.^27^ Furthermore, women are significantly more likely than men to believe that self‐injury serves solely for self‐stimulation, despite evidence indicating multiple functions.^18^ Contrary to studies on suicide myths, men did not consistently show a higher propensity to believe self‐injury myths as compared to women. The findings suggest that some myths are more readily accepted by women, depending on their content. However, this study did not find a significant trend of consistent beliefs in all myths by either gender. The reasons for the observed difference between men and women could be complex and multifaceted, and further research is needed to clarify the underlying factors.

Higher myth endorsement exists among those aged older than 60 years,^13^ potentially due to cohort effects in mental health awareness. Conversely, our results reveal that belief in the myth of self‐injury did not vary as significantly across age groups compared to gender differences. The myth that “self‐injury is exclusively undertaken for stimulation” was significantly less believed across other age groups, with 20‐year‐olds as the reference group. This finding may indicate that middle‐aged and older individuals are more inclined than younger age groups to view stimulus seeking as inapplicable motivation for self‐injury, or they consider self‐injury to serve other functions, such as emotion regulation. Furthermore, regarding certain myths, such as “self‐injury is driven by the desire to be cared about; therefore, it will decrease if ignored” and “talking about self‐injury encourages it,” we observed a tendency for the younger demographic to exhibit a higher belief in these myths compared to the middle‐aged group. However, none of the myths demonstrated an inverse relationship with the older group.

Several potential factors may contribute to the higher prevalence of certain myths among individuals in their 20s than among older age groups. Younger individuals may experience increased exposure to misinformation on mental health and self‐injury through social media platforms and other online sources. Furthermore, younger individuals may be more susceptible to influence from peers, who may hold these misconceptions. For instance, the myth that “talking about self‐injury encourages it” may be more prevalent among young people because of a lack of open communication about mental health. They may harbor concerns that addressing self‐injury could lead to further harm. Notably, self‐injury is a common phenomenon among young people; however, this does not necessarily indicate that individuals of the same age group possess a comprehensive understanding of the factors and functions underlying self‐injury. Nevertheless, since young people do not uniformly believe in all myths, targeted interventions are necessary for psychoeducation and social awareness initiatives that consider the specific beliefs of each age group regarding myths and their underlying factors.

Individuals with experience in human‐service professions demonstrated a significantly lower likelihood than those without such experience of perceiving self‐injury solely as a symptom of mental illness. Indeed, self‐injury is not necessarily exclusively committed by individuals with mental illness. Those with a background in human‐service professions demonstrated a greater propensity to reject this misconception compared to those without such a background, suggesting a more accurate comprehension of the phenomenon. Nevertheless, among the 14 myths examined, only this particular myth demonstrated a significant association in the direction of myth rejection when comparing individuals with a background in human‐service professions to those without such a background. Notably, despite the multifaceted nature of self‐injury functions, a significantly higher proportion of individuals in human‐service professions perceived self‐injury as an attention‐seeking behavior. Self‐injurious behaviors can elicit intense emotional responses in professionals, including frustration and helplessness. These affective reactions may contribute to the perception that a behavior is manipulative. Furthermore, professionals may not fully comprehend the complex etiology of self‐injury and may resort to stereotypical and reductionist explanations. The direct association of self‐injury with borderline personality disorder may also lead to the interpretation that self‐injury is primarily intended to attract attention from others in an individual's environment. Consequently, these findings suggest that professional experience in human services does not necessarily dispel misperceptions about self‐injury. Enhancing psychoeducation for human‐service professionals based on the current results is valuable for mental health and suicide prevention policies. Presenting research that highlights the diverse functions of self‐injury can help clarify its background to professionals. In the context of training, it is imperative to address the misconceptions of professionals through constructive discourse, rather than attempting to rectify these misconceptions unilaterally and expeditiously.

Notably, participants who reported greater confidence in their ability to address self‐injury appropriately were more likely to endorse false beliefs. This seemingly paradoxical result suggests that individuals who perceive themselves as well‐informed about self‐injury may be prone to providing incorrect responses, highlighting the need for intervention. One potential explanation for this result is the Dunning–Krueger effect: a cognitive bias in which individuals overestimate their abilities,^28^ consequently leading to suboptimal decision‐making. Although not focused on self‐injury, previous research has demonstrated that individuals with low health literacy reported equivalent or higher confidence in their health knowledge compared to those with high health literacy and that individuals with lower health literacy experienced more difficulties engaging in health‐promoting behaviors.^29^ Individuals with high confidence levels may exhibit reduced receptivity to novel information or alternative perspectives. Owing to their belief in possessing sufficient knowledge of a subject, they may dismiss or minimize evidence that contradicts their established beliefs. Such confirmation bias could reinforce erroneous beliefs over time. Confident individuals may demonstrate a preference for simple explanations of complex phenomena. Myths and misconceptions often provide explanations that are more appealing than the nuanced reality; this may apply to self‐injury. Confidence in addressing self‐injury may also stem from personal experience and informal, rather than formal, training. In the absence of a proper education, these individuals may rely on myths and misconceptions. This finding suggests that well‐intentioned efforts by the public to intervene in self‐injury may be predicated on biased information; consequently, there exists a potential risk that such efforts may prove counterproductive to individuals engaging in self‐injurious behaviors. Therefore, additional efforts are warranted to enhance awareness and promote psychological education. For instance, in educational settings, the finding that individuals with greater confidence in responding to self‐injurious behavior are more likely to endorse myths underscores the importance of promoting evidence‐based interventions rather than relying on self‐serving assumptions. Facilitators of such training must refrain from imposing predetermined responses and instead foster an environment conducive to expanding perspectives through critical self‐reflection and participant discourse. These findings are valuable for introductory training and educational materials.

Finally, the findings indicated that the connection between aversion to self‐injury and incorrect beliefs is robust and pervasive. While it is impossible to determine which precedes the other, it is likely that stigma increases the likelihood of holding inaccurate beliefs and that misperceptions about self‐injury simultaneously contribute to stigmatization. These results suggest that simply providing accurate information is insufficient for public awareness campaigns aimed at creating a supportive atmosphere for individuals with self‐injury. To foster a rational, evidence‐based response, it is essential to provide comprehensive psychological education that considers people's irrational feelings and cognitive biases.

## LIMITATIONS

This study had some limitations. First, participants were recruited using convenience sampling from a web‐based research company's registry, introducing potential bias owing to using a non‐representative sample. Measures were taken to exclude participants lacking cognitive resources, but the enrollment process itself may have caused selection bias. For example, individuals in their 60s who have registered with an online research platform may exhibit enhanced digital literacy or intellectual curiosity compared to the general population within the same age cohort, making them less representative of the general population. Future studies should use probability‐sampling techniques to address this limitation.

Second, the methodologies employed to identify myths regarding self‐injury in this study were not consistently adequate. There is no standardized measure for myths about self‐injury; therefore, the researchers identified myths based on a literature review, selecting items inconsistent with empirical data and statistics. The validity of these items remains uncertain, and participants could have interpreted the myths differently than intended by the authors. Future studies should refine methodologies for accurately identifying myths.

Third, this study did not include all sociodemographic factors and personal experiences potentially associated with self‐injury myths. Lower education levels are associated with lower suicide literacy,^14^ which may relate to self‐injury myths; however, these factors were not considered here. Future research should incorporate a broader range of variables, such as participants' personal history of self‐injury and suicide attempts, to investigate this issue more comprehensively.

## CONCLUSION

Our findings provide novel insights into suicide and self‐injury prevention strategies and interventions. The intricate relationship among demographics, personal experiences, and beliefs embedded in self‐injury myths is influenced by a multitude of factors, and further exploration is necessary to comprehensively understand their complex interactions. Moreover, recognizing the prevalent misconceptions among the public is crucial for effective information dissemination and enhanced psychoeducation. Avoiding stereotypes and generalizations related to self‐injury and providing accurate information is vital to fostering understanding and support.

## AUTHOR CONTRIBUTIONS

Masaru Takahashi and Izumi Kadomodo designed the study, the main conceptual ideas, and a proof outline. Masaru Takahashi collected the data. Izumi Kadomodo, Kasumi Imahara, Kayoko Myojo, Michiko Yasuda, and Yukiko Miyamoto aided in interpreting the results and reviewed the manuscript. Masaru Takahashi supervised the project. Masaru Takahashi wrote the manuscript with the support of Izumi Kadomodo, Kasumi Imahara, Kayoko Myojo, Michiko Yasuda, and Yukiko Miyamoto. All authors have reviewed the results and approved the final version of the manuscript.

## CONFLICT OF INTEREST STATEMENT

The authors declare no conflict of interest.

## ETHICS APPROVAL STATEMENT

This study was approved by the Humanities and Social Sciences Research Ethics Committee of the Ochanomizu University (No. 2023‐135).

## PATIENT CONSENT STATEMENT

Informed consent was obtained from all participants. The beginning of the survey form clearly stated that participation was voluntary, that no one would be disadvantaged if they did not participate, that they did not have to answer questions that were difficult to answer, and that they could withdraw their consent at any time.

## CLINICAL TRIAL REGISTRATION

N/A.

## Data Availability

The datasets generated and/or analyzed during the current study are available from the corresponding author upon reasonable request.
